# Novel Biomarkers for the diagnosis of diabetic nephropathy 

**DOI:** 10.22088/cjim.15.3.382

**Published:** 2024-08-01

**Authors:** Marcio Concepción, Juan Quiroz, Jacsel Suarez, José Paz, Pela Roseboom, Sofia Ildefonso, Denis Cribilleros, Francisca Zavaleta, Julia Coronado, Luis Concepción

**Affiliations:** 1 Universidad Científica del Sur, Lima, Peru; 2 Division of Medicine, Hospital de Apoyo Chepén, Peru; 3 Division of Endocrinology, Edgardo Rebagliati Martins National Hospital, Lima, Peru; 4 Faculty of Medicine, San Marcos Mayor National University, Lima, Peru; 5 Department of Medicine. Cesar Vallejo Mendoza Hospital, Santiago de Chuco, Peru; 6 Division of Endocrinology, Guillermo Almenara Irigoyen National Hospital, Lima, Peru; 7 Division of Nephrology, National Center of Renal Heath, Lima, Peru; 8 Division of Neonatology. Belén Hospital of Trujillo, Trujillo, Peru; 9 Clínica Internacional, Lima, Peru; 10 Department of Medicine. Hospital Regional Docente of Trujillo, Trujillo, Peru; 11 Faculty of Medicine, National University of Trujillo, Trujillo, Peru

**Keywords:** Biomarkers, Diabetic nephropathy, Diabetic kidney disease, Diagnosis, Quality of life

## Abstract

Diabetes mellitus and its complications are a known public health problem nowadays. Diabetic nephropathy is one of the main complications and the result of multiple mechanisms, including: activation of the renin-angiotensin-aldosterone system, formation of advanced glycation end products and chronic inflammation that led to glomerular and tubulo-interstitial damage producing mesangial expansion and glomerulosclerosis, which finally results in chronic kidney disease. Early detection of diabetic nephropathy is essential for adequate intervention to stop, or at least slow down its progression. Multiple markers have been described, not only the classic ones such as serum creatinine, urea, and albuminuria, but at this point also novel biomarkers such as neutrophil gelatinase-associated lipocalin, tumor necrosis factor 1 receptor and monocyte chemoattractant protein-1, among others. The aim of this article was to provide an update review of the role of biomarkers in the diagnosis of diabetic nephropathy.

Diabetes mellitus (DM) has been a chronic disease with an increasing prevalence in recent years (1). Currently, according to the International Diabetes Federation, over 537 million people have DM (1, 2). It is considered a serious public health problem because of its impact on quality of life and associated health costs (3). Among its chronic complications, one of the most frequent is diabetic nephropathy (DN), which is associated with significant morbidity and mortality, and is the leading cause of end-stage renal disease (ESRD) (4, 5). DN is characterized by abnormal albumin excretion in the urine with or without a reduced glomerular filtration rate (GFR) (6). This occurs in 20-40% of patients with DM (7). Between 23 and 36% of type 1 DM (T1D) patients will develop albuminuria, a situation that also occurs in around 38% of those with type 2 DM (T2D) (8).

The main modifiable risk factors are increased urine albumin excretion, (6) hyperglycemia, high blood pressure, dyslipidemia, obesity, and smoking (6, 9). The non-modifiable risk factors are advanced age, female gender, and the duration of DM (9). 

DN in its early diagnosis and timely intervention in diabetic patients are important to slow down the progression of renal function decline and prevent ESRD (10).

The aim of this narrative review was to update the usefulness of serum and urinary biomarkers in the diagnosis of DN, and its clinical relevance is to improve the management of DN and to limit its impact on the morbimortality which will result in maintaining a better quality of life for the patient and lower health system costs for this chronic disease. 

## Methods

**Search strategy:** MEDLINE and EMBASES electronic databases were searched for completed studies of any design except case reports, case series, letters to the editor and conference proceedings, from database inception between 2005 and 2022. The Medical Subject Heading (MeSH) used were "diabetic kidney disease" or “diabetic nephropathy”, and “biomarkers”.

**Inclusion and exclusion criteria:** Inclusion criteria were studies published in English involving patients of any age. Systematic reviews, clinical trials, prospective cohort studies, cross-sectional and retrospective studies, and narrative reviews related to the objective of this manuscript were included. The investigation was limited to articles related to human beings, so exclusion criteria was non-human studies.

**Figure 1:**
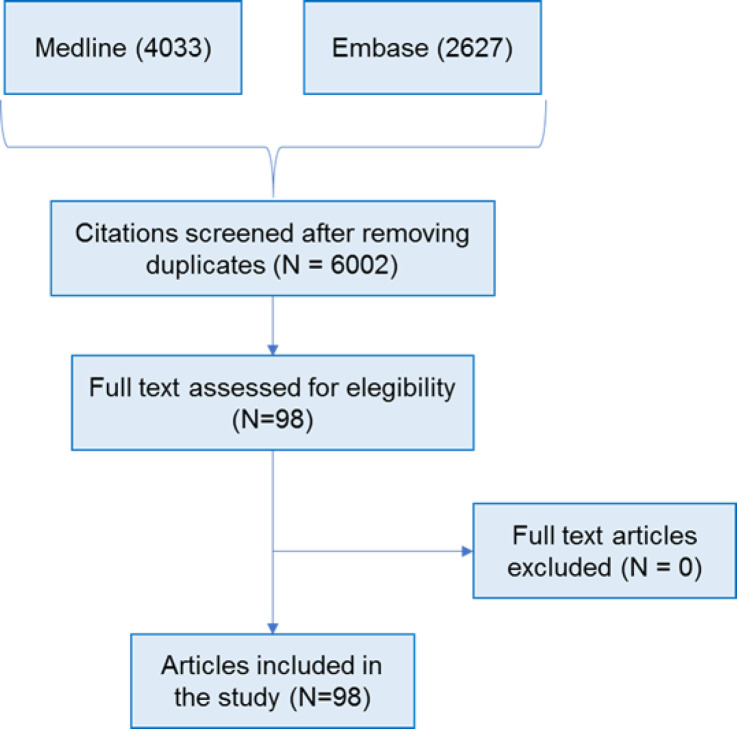
Flowchart of narrative review process

**Screening:** A total of 6660 citations were identified; 658 duplicates were removed; 6002 titles and abstracts were screened against eligibility criteria. No information produced outside of traditional publishing and literature distribution channels was included. 5890 titles were excluded at the title and abstract screen, 98 eligible full-text papers met the inclusion criteria. This process is summarized in figure 1.

**Data extraction and synthesis:** Results from the included papers were extracted to a table about pathophysiology, diagnosis, traditional and novel biomarkers.

**Quality assessment:** The quality of this narrative review was evaluated using the SANRA scale, obtaining 12 points (11).

## Results

**Figure 2:**
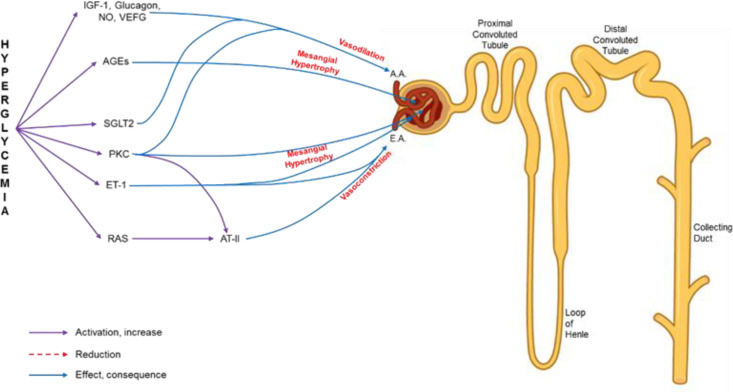
Representative diagram of the nephron and the alterations that contribute to diabetic nephropathy. A.A.: afferent arteriole; E.A.: efferent arteriole; ET-1: endothelin 1; RAS: renin angiotensin system; AT-II: angiotensin II; IGF-1: insulin-like growth factor 1; NO: nitric oxide; VEFG: vascular endothelial growth factor; AGEs: advanced glycation end products; PKC: protein kinase C; SGLT-2: type 2 sodium-glucose co-transporter.

**Pathophysiology of diabetic nephropathy:** Hyperglycemia is currently recognized as the initial mediator of metabolic and hemodynamic alterations in the kidney, associated with other pathophysiological events, (12, 13) summarized in figure 2, and as follows: 

**Hemodynamic alterations:** Activation of the renin-angiotensin system (RAS) increases the level of angiotensin II, produces contraction of the efferent arteriole, and increases intraglomerular pressure, resulting in hyperfiltration and damage to the glomerular basement membrane (GBM) (14-18).

**Metabolic alterations:** Hyperglycemia leads to increased glycolysis by stimulating the polyol and hexosamine pathways, formation of advanced glycation end products (AGEs), and activation of protein kinase C (PKC) (13, 15).

AGEs cause cell damage by altering the function of laminin and type IV collagen, increasing the permeability of the GBM (6, 19-21). Additionally, they bind to proinflammatory receptors, which activate the production of interleukin (IL) 1 and 6, tumor necrosis factor alpha (TNF-α), transforming growth factor β1 (TGF-β1), vascular endothelial growth factor (VEGF), and reactive oxygen species (19, 22, 23).

On the other hand, PKC contributes to DN by increasing levels of prostaglandin E2 and nitric oxide, leading to vasodilation of the afferent arteriole and enhances the action of angiotensin II in the efferent arteriole, favoring the development of glomerular hyperfiltration (24-26). Also, this stimulates the production of fibronectin and type IV collagen, resulting in thickening of the GBM (27). Hyperglycemia itself causes dilation of the afferent arteriole through the release of vasoactive mediators such as insulin-like growth factor 1, glucagon, VEGF and prostaglandins (28).

**Inflammatory changes:** To sum up contributions to the development of DN, diabetic patients suffer chronic activation of innate immunity and a proinflammatory state (29). The nuclear factor kappa-light-chain-enhancer of activated B cells (NF-κB) regulates the expression of genes related to inflammation and apoptosis (30). Hyperglycemia increases the NF-κB expression through activation of Toll-like receptors (17, 31-33). In DN, NF-κB activation is associated with proteinuria and interstitial cell infiltrate (30, 34, 35).

Inflammatory cytokines such as TNF-α and IL 1, 6 and 18 are expressed in higher amounts in kidneys of patients with DM. These cytokines correlate directly with the degree of albuminuria and possibly increase vascular endothelial cell permeability, contributing to glomerular hypercellularity and thickening of the GBM (36-39).

Hyperglycemia increases the expression of the sodium-glucose cotransporter 2 (SGLT-2), which, in contexts of DM, is counterproductive (40, 41). This causes hemodynamic and metabolic alterations, increasing sodium and glucose reabsorption, stimulating the macula densa of the distal tubule, causing dilation of the afferent arteriole by tubulo-glomerular feedback, and increasing further intraglomerular pressure (42-46).

## Diagnosis:

**Traditional biomarkers:**

**Creatinine and estimated GFR:** Serum creatinine is the most widely used biomarker to assess kidney function; it corresponds to the end-product of the creatine and creatine phosphate metabolism. Anyhow, creatinine measurement is prone to interferences, and inaccuracies due to physiological and technological aspects. 

Its association with muscle mass, tubular secretion, diet, and comorbidities such as advanced liver disease, cause its clinical limitations. So does extrarenal clearance of serum creatinine possibly due to intestinal bacteria, relevant in advanced CKD (47) The serum creatinine concentration starts to increase when approximately 40–50% of the kidney parenchyma is damaged (48, 49). This limits its use as a marker for the early diagnosis of CKD (50). Different formulas used to estimate the GFR based on serum creatinine level are widely used, but they lack of accuracy since they do not measure direct renal tissue injury and have a low sensitivity to small changes in renal function (51-53). Additionally, there are differences in the estimation formulas of the GFR based on serum creatinine (eGFRcr), which limits, even more, its utility (54).

**Albuminuria:** Albuminuria is a traditional biomarker for DN. Albumin is almost completely reabsorbed in the renal tubules. The urinary albumin-creatinine ratio (UACR) is the method of choice for detecting albuminuria using a simple urine sample. Albuminuria, defined as a urine albumin excretion equal to or greater than 30 mg/day, is considered a sign of glomerulo-tubulopathy, which correlates with renal structural changes (47). It is the strongest predictor of kidney disease and cardiovascular morbimortality (55, 56). 

However, prognostic by albuminuria is not specific for DN. Approximately 30% of patients with DN do not have albuminuria and therefore, eGFR is a better biomarker in the prediction of DN development and its progression (57-59). Studies show that patients with T2D without albuminuria have a high risk of renal dysfunction progression due to inflammatory mechanisms and lesions at the level of the proximal renal tubule, through alteration of tubuloglomerular feedback (60). The characterization of non-albuminuric forms of DN reinforces the importance of adding the estimated GFR to the measurement of albuminuria (59). GFR and albuminuria are independent predictors of the course of kidney disease and risk of mortality, and therefore both should be evaluated in screening for DN (61).

**Cystatin C:** Cystatin C is a low molecular weight protein, which concentration correlates with GFR. Its superiority over other renal function markers is due to its ability to remain free of binding proteins to be filtered in the glomeruli and to be almost completely reabsorbed in the proximal convoluted tubule. It has less interindividual variation, as it is independent of muscle mass, gender, age, and inflammatory conditions (62). 

The estimation GFR based on serum Cystatin C (eGFRcys) has been suggested to show better clinical utility for detecting nephropathy in patients with normoalbuminuria, as well as predicting the progression of nephropathy in patients with albuminuria (63). Studies suggest that serum cystatin C could increase before creatinine in the presence or progression of DN, but its significance in DN is still on debate since there is no high-grade evidence to recommend it as a screening method for DN (64-68).

 Additionally, urinary excretion of Cystatin C is elevated early in diabetes and prediabetic nephropathy and it suggests tubular injury (55). 

**New biomarkers: **Current research is aiming to find more sensitive, specific, and precise biomarkers that allow the early detection of kidney damage, even before the appearance of microalbuminuria or a GFR decrease. New biomarkers initially found in acute kidney injury, are being studied to determine their value in the evaluation of CKD (51). According to the classification of sample origin, biomarkers are urinary or serum (table 1); meanwhile according to the site of injury, there are glomerular and tubular biomarkers (table 2). Pathophysiological mechanism classification considers renal dysfunction, inflammation, and oxidative stress biomarkers (55).

**Table 1:** Classification of new biomarkers according to sample origin

**Urinary biomarkers**	**Serum biomarkers**
Alpha-1 microglobulin Ceruloplasmin Cyclophilin A IgG KIM-1 MCP-1 Megalin NGAL 8-oxo-7,8-dihydro-2-deoxyguanosine TNF-alpha Transferrin Type IV collagen VDBP	NGAL NT-proBNP TNFR1 and TNFR2 Uric acid

KIM-1: Kidney injury molecule 1; MCP-1: Monocyte chemoattractant protein-1; NGAL: neutrophil gelatinase-associated lipocalin; NT-proBNP: N-Terminal B-type natriuretic peptide prohormone; TNF-alpha: Tumor necrosis factor ALPHA; TNFR1: Tumor necrosis factor 1 receptor; TNFR2: Tumor necrosis factor 2 receptor; VDBP: vitamin D binding protein.

**Table 2:** Classification of new biomarkers according to place of lesion

**Glomerular biomarkers**	**Tubular biomarkers**
Ceruloplasmin IgG Transferrin Type IV collagen TNFR1 and TNFR2	KIM-1 NGAL Megalin Alpha-1 microglobulin VDBP

## Tubular biomarkers:

**Neutrophil gelatinase-associated lipocalin (NGAL):** NGAL is a low molecular weight glycoprotein, a constituent of neutrophil granules that also is expressed in the kidney. This protein is freely filtered by the glomerulus and reabsorbed in the proximal tubules. When epithelial tubular cells of the kidneys are acutely injured, there is a marked increase in urinary NGAL concentrations and might go with an increase in *de *novo synthesis of NGAL within the tubules (62, 69).

Although an increase in urinary as well as serum NGAL has shown to be a reliable marker for the early diagnosis of acute kidney injury, its association with CKD progression remains controversial. It correlates inversely with GFR, but does not correlate with HbA1c. At the same time, T1D and T2D patients with normoalbuminuria can present elevated values of urinary NGAL, predicting albuminuria (65, 69). Its sensibility and specificity were reported to be 94% and 90%, respectively (70).

**Kidney injury molecule 1 (KIM-1):** KIM-1 is a glycoprotein found in the proximal tubules and is a sensitive marker of proximal tubular injury. Increased levels of KIM-1 have been found in DM patients, independently of albuminuria. KIM-1 has been associated with DN progression (71). With a cut-off point of 32.00 ng/g and sensitivity 93.8% it’s reported sensibility and specificity are 93.8% and 88.5%, respectively (72).

**Vitamin D binding protein (VDBP):** VDBP is the main plasmatic transporter of vitamin D. It is vital in the biosynthesis of 1,25 dihydroxy-vitamin D within the renal proximal tubules, binding 25-hydroxy-vitamin D to VDBP and actively recovering the complex by endocytosis from the glomerular filtrate. Higher expression of VDBP was found in patients with DN, although the reasons remain unclear. It is suggested that high concentrations are associated with renal tubular damage as well low serum levels of vitamin D, which has also been found a factor associated with DN. This marker has a sensitivity of 98.8% and a specificity of 80%, for a cut-off point of 216 ng/mg (73).

**Megalin**: Megalin is a multiligand receptor expressed in proximal tubular cells that reabsorbs filtered albumin and correlates cross-sectional with albuminuria (74). There are 2 types of assays: A-megalin, which is elevated in patients with early DN and microalbuminuria (75), and C-megalin, which is associated with persistent microalbuminuria and useful specially in patients with low-normal UACR levels (74). 

**Alpha-1 microglobulin:** Urinary alpha-1 microglobulin is reabsorbed in the proximal tubule. It is an early biomarker of tubular injury that predicts microalbuminuria in T2D patients (76).

## Glomerular biomarkers:

**Transferrin:** Transferrin is an iron-binding protein with low molecular weight and low ionic load that easily crosses the glomerular barrier (77). Urinary transferrin appears elevated prior to the development of microalbuminuria in patients with DM, suggesting that it may be an early biomarker of glomerular damage and could predict microalbuminuria (78, 79).

**Ceruloplasmin:** Ceruloplasmin is a copper-carrying protein that crosses the glomerular barrier with difficulty due to its negative load. Elevated urine ceruloplasmin levels usually predict microalbuminuria in T2D patients with normoalbuminuria (78-80). 

**IgG:** Urinary IgG is a biomarker of glomerular damage that usually appears elevated along with transferrin and ceruloplasmin and predicts microalbuminuria in patients with DM (78, 79).

**Type IV Collagen:** Type IV collagen is the principal component of the glomerular basement membrane and mesangial matrix and is present in podocytes and the proximal tubule. Hyperglycemia stimulates the synthesis of type IV collagen. Urinary type IV collagen is a structural damage biomarker and its level increases as DN progresses (81, 82).

**Tumor necrosis factor 1 receptor (TNFR1) and Tumor necrosis factor 2 receptor (TNFR2):** Elevated serum TNFR1 and TNFR2 concentrations are strong independent predictors of renal function decline leading to ESRD in patients with T2D. Elevated serum concentrations of TNFR1 or TNFR2 in T2D are associated with early glomerular structural lesions. These receptors showed the strongest associations with a reduced percentage of fenestrated endothelium (83).

## Inflammation biomarkers:

**Monocyte chemoattractant protein-1 (MCP-1):** MCP-1 is a proinflammatory cytokine that plays a role in the recruitment of macrophages and monocytes, being detectable in urine of patients with different kidney diseases. A significant increase in MCP-1 levels has been found in urine of T2D patients with macroalbuminuria. Its measurement would especially be useful to determine the prognosis of DN (84, 85).

**Tumor Necrosis Factor Alpha (TNF-α):** TNF-α is a cytokine involved in systemic inflammation. It is produced primarily by activated macrophages. Urinary excretion of TNF-α is increased in T2D patients with microalbuminuria and macroalbuminuria compared to those without albuminuria (86).

**Biomarkers of oxidative stress:**

**Urinary 8-oxo-7,8-dihydro-2-deoxyguanosine (8-oHdG):** Oxidative DNA damage causes the production of 8-oHdG, which is eliminated in the urine and so considered a biomarker of oxidative stress. Higher levels of 8-oHdG in urine indicate significant progression of DN (82). Eventually, this biomarker might predict long-term mortality in DM (87).

## Other biomarkers:

**B-type natriuretic peptide prohormone (NT-proBNP):** The BEAt-DKD consortium, which assessed biomarkers to predict the rate of estimated GFR reduction in the early stages of CKD in patients with DM, showed that NT-proBNP predicts reduction of estimated GFR (88).

**Periostin:** Periostin is a urinary biomarker with high diagnostic precision in DN. Periostin reaches a sensitivity of 98% and a specificity of 80%. It is useful for early detection of DN (89, 90).

**Cyclophilin A:** Cyclophilin A is used as a urinary biomarker for early detection of DN. It has a high diagnostic precision in DN and registers a sensitivity of 84% and a specificity of 86% (89, 90).

**Uric acid:** Serum uric acid is associated with insulin resistance and endothelial dysfunction. Uric acid increases in chronic kidney disease and is associated with GFR reduction.

Also, it predicts microalbuminuria in T1D patients, being an early biomarker of DN (91, 92).

**Proteomic:** Proteomics is the large-scale study of proteins, which allows the identification and quantification of hundreds of proteins or peptides, and their variation under conditions of health or illness (93). In recent years, proteomics has become a promising method for identifying a wide variety of kidney disease biomarkers in urine, which is easily accessible, a very stable medium for proteins, and non-invasive for the patient (94).

The PRIORITY trial (Proteomic Prediction and Renin Angiotensin Aldosterone System Inhibition Prevention of Early Diabetic Nephropathy in Type 2 Diabetic Patients with Normoalbuminuria) confirmed that the test assessing a panel of 273 proteins and peptides using mass spectrometry, known as CKD273 (CKD classifier 273), is useful in the determination of the risk for DN progression (95).

**MicroRNA:** MicroRNAs are relatively stable particles and can be measured in serum, plasma, urine, and saliva by polymerase chain reaction, microarrays, RNA sequencing, and in situ hybridization. The microRNAs miR-192-5p and miR-130b have been identified and could be useful in the early detection, monitoring, and treatment efficacy of CKD (96).

**Lipidomics analysis:** Lipidomics is the large-scale study of pathways and networks of lipids. Alterations in lysophosphatidylethanolamine, phosphatidylethanolamine and triacylglycerol may be associated with alterations in the lipid metabolism in DN and the severity of the development of nephropathy, being able to identify early from advanced damage (97).

**Conclusion:** DN results in multiple alterations at glomerular and tubulo-interstitial level caused by hyperglycemia, hemodynamic factors, oxidative stress, and underlying chronic inflammation. These alterations can be detected earlier by the novel biomarkers, allowing timely diagnosis and treatment, to stop or slow down glomerular and tubular damage, thus reducing morbidity and mortality and so, maintaining a better quality of life for the patient, and reducing health system costs. 
